# Electricity in the Treatment of Pulpless Teeth

**Published:** 1898-04

**Authors:** Chas H. Rosenthal

**Affiliations:** Cincinnati, Ohio


### Electricity in the Treatment of Pulpless Teeth.

BY CHAS H. ROSENTHAL, D.D.S., CINCINNATI, OHIO.

Read before the Cincinnati Academy of Dentistry.

Since the first experiments of Galvani, in 1786, to the present time, the strides of the practical uses of electricity have been the marvel of civilization. Although we are ignorant of the exact nature of electricity, we are by no means ignorant of the laws that control it. The devices of human ingenuity have placed methods at our hand by which we can produce like causes by like effects. We touch a key, and instantly we encircle the world. The dynamo and motor are made to do our bidding, from the largest to the smallest quantity of electro-motive force; all are under our control. This has given it a place in the healing art, electro-therapeutics.

Its use in dental therapeutics is interesting to us. Let us consider one of its uses:

THE TREATMENT OF PULPLESS TEETH.

The method I am about to present, I desire to say, is an original one, and can, therefore, only give you the results of my own experience.

Let us consider first some of the elementary principles employed:

ELECTRIC OSMOSIS, OR CATAPHORESIS AND ELECTROLYSIS.

From a medical standpoint we understand by osmosis, or cataphoresis, the introduction of medicaments by means of electricity into the body through skin, mucous membrane, or teeth with enamel removed.

Electrolysis is the decomposition of chemical compounds of certain kinds, known as electrolytes. Chemical energy can be converted into electric energy, so does electric energy convert chemical energy. Where these two principles are used combinedly, they are known as "metallic electricity"—the first, no doubt, being a mechanical force. The binary electrolytes, that is to say, compounds containing but two elements with an electro-positive and electro-negative, are easiest decomposed.

The advantages of the employment of binary compounds in dental therapeutics is apparent, since it requires but a low amperage to decompose them. We have also to deal with secondary reactions, chemical compounds formed by contact with other elements present for which they have a chemical affinity. The products of the decomposition are known as ions, respectively cathode and anode, depending upon their affinity for either upward or downward pole.

Let us apply the principles of metallic electricity to the treatment of a pulpless tooth and note its therapeutical effect, taking, for instance, a putrid pulp, one where the canals are open, with no peridental membrane inflammation. We will start by carefully applying the rubber dam, being cautious to leave no contact with neighboring tissue. This done, thoroughly wash out the pulp canal with a 6 percent hydrogen peroxide ($H_2O_2$) ridding the canal of much of the putrid tissue. A saturated solution of sodium chloride (Na C) is now carried to the pulp canal on a platinum electrode, using the positive pole of a galvanic current. (It is important that the electrode be platinum on account of the secondary reaction consequent to the use of other metals.) Not more than one-fourth to one milliampere of current is necessary to decompose this binary compound; the decomposition commences at once.

The secondary reaction at this point is important. We obtain as a result, not chlorine and sodium, but chlorine and hydrogen set free, because the sodium, primarily formed, decomposes the water $H_2O_2$, forming NaHO (caustic soda). We have, therefore, not only the ion chlorine, with its powerful antiseptic effect in its nascent state, but also the caustic soda as a detergent by cataphoresis these products are carried completely through the tooth, reaching everything contained within the pulp canal.

The success of this treatment depends entirely upon the time it is allowed to remain in the tooth; germicides are only effective when allowed to remain in contact with germs sufficient time to destroy them, and so with this, it requires about twenty-five to thirty minutes to accomplish desired results.

Removing the electrode, allowing whatever moisture present to remain, apply a two percent solution of nitrate of silver to the pulp canal and there will be an immediate precipitate of chloride of silver by its contact with the remaining chloride, this insoluble salt is deposited to the apex of the tooth and serves as a root-canal filling, it is unnecessary to do anything further; the tooth can be filled at once. I have used this treatment in several hundred cases, extending over two years, with comparatively few failures, and those were recorded in my early experience, when I did not recognize the importance of leaving the application a sufficient time. Where there is an inflamed peridental membrane the pulp canal should be opened up to afford drainage and allow the tooth to become free from soreness; that is to say, the membranes should be free from inflammation, an electric current takes the shortest course from anode to cathode, and, as a consequence, if there is any disturbance at the side of the root, it would not be reached by cataphoresis. The tooth once free from soreness, proceed as in the first instance. Of the cases where fistula is established, incidentally I wish to say, I can suggest no better method than the time-honored injection of carbolic acid through the root canals.

DISCUSSION.

Dr. N. H. Grove: May I ask, do you use the 110-volt current reduced down, or otherwise?

Dr. Rosenthal: So far as that is concerned, it does not make any difference what current is used, provided you have a controller and milliampmeter. In the discussions as to the use of street currents as against battery currents, there is this to say: In my office I get the current directly from the dynamo in the building, and am not troubled with heavy electric currents which may cause great shock, or burning out of wires, etc., so it does not make any difference so that the current is controlled, whether it is produced from dry or wet cells, from a dynamo or in whatever way it is produced; if it is a milliampere, it is a milliampere, just the same.

The subject of electricity is very vaguely understood, I think, in a general way by the practitioners, and many of them have very curious ideas with reference to it, as though there were different kinds; it is always the same. If we can control it, it is always the same. A current not interrupted is always the same under all circumstances. As I have said, like effects produce like results uniformly.

Dr. W. T. McLean: My knowledge of electricity in its application to dental work is quite limited. Dr. Rosenthal's paper is quite interesting, and as he obtained satisfactory results for two years this is alone sufficient to commend it as being applicable to dental work. There is one thing I failed to glean from the paper, and that is the application of the current in putrid canals for the purpose of eliminating all the suppurative material that may be there; I did not quite grasp that. And with reference to the medicament used and the method of controlling the amount supplied to the various teeth, that is something I am not really competent to discuss at all, because I do not understand it. It occurs to me, however, that having the ability to control the electric current is really the bugbear, and as the Doctor says he can control it, irrespective of the physical condition of the patient or teeth, I suppose it is dependent upon experience, of course. There is no question, I think, but that electrical osmosis is in its infancy as yet, and I think the time is approaching when we will derive great benefits from it. The quality of the current used, Dr. Rosenthal claims, is always the same. Of course, an apparatus where you have a milliampmeter, or something to control the electrical current, is very necessary. I believe the great physical differences in the constitutions of the persons operated upon may have something to do with the effect. Some patients would not stand such heroic treatment in the extirpation of suppurative pulps, where others would take a great deal of this treatment without fazing them. However, I am really not able to discuss the paper, having heard it read but the one time.

Dr. Rosenthal: You may divorce electrolysis from osmosis; they are two different forces. The force of cataphoresis or osmosis is a mechanical one; it is simply the ability of the electric current to carry before it a medicament that has an electro-positive or electro-negative of carrying that through the tooth to the electrode of the positive side. Electrolysis is the decomposition of chemicals; now, it does not make any difference whether your patient can stand much or little electricity, because this operation in a devitalized tooth is painless where you use from one-quarter to one milliampere. You might have a milliampmeter that would register three milliamperes, but you must be conversant with your own machine to understand about how much current it takes to decompose these elements. In the decomposition of chloride of sodium, it depends upon how much electro-motive force an element will make, as to how much electro-motive force it will take to decompose it. You can not make much electro-motive force with the chloride of sodium, and consequently one-fourth milliampere is all that is necessary. If you use too much, you get no effect at all. The current is so weak that you are just able to register that amount of current on a very fine milliampmeter, so that if a man has no milliampmeter at all, he can simply, by experience, tell about how much current to use; use it until the patient just about feels it, and then turn it about one-half way back. It produces quite a quantity of chlorine, and so long as you are getting the chlorine, and not hurting the patient, you have neither too much nor too little. As to the resistance of patients, that makes no difference in the measurement of electro-motive force employed; the resistance is not counted in the meter at all.

So far as the dial of the apparatus is concerned, you may have to turn that away around in some individual's hands, where the skin of the hand is very callous, the contact being bad, so that, as to the amount of current actually used, it makes no difference on the controller how far you throw it around, as that amount of current does not pass through the patient, and so you must have a milliampmeter. This latter may not register correctly, but if you become familiar with it and get the practical results, there is no difference if milliampmeters do vary.

They are not made uniform; one manufacturer makes them to register higher, the other lower.

As to how I rid the canals of putrescent matter: I wash the canals out with dioxide of hydrogen, ($H_2O_2$) which rids them of much of the putrid matter. It would not really make much difference if that were left in, as I have tried it, leaving the *debris* in at times, simply sterilizing it. Nascent chlorine is about as good an antiseptic as therapeutics afford.

Dr. McLean: As to the force generated from the battery, how do you determine the fact of there not being forced through the apical foramen putrid matter which may not be sufficiently disinfected?

Dr. Rosenthal: But one element can be carried from electro-positive to electro-negative on the principle of cataphoresis. Organic matter will not be affected; organic matter will be decomposed, but not affected by cataphoresis. You can not force organic matter through a tooth or through anything; it is against all the principles of electrical osmosis. You simply carry your chlorine through, by reason of its having an affinity, from positive to negative, so it only carries the element chlorine through; there is no question about that. An experiment, for instance, that I have made: If the electrode is intercepted by a solution of nitrate of silver and chloride of silver, notwithstanding there is no chlorine on the point placed in the solution of chloride of silver, a decomposition takes place, making the chloride of silver from the nitrate. This proves conclusively that the chlorine is carried through. Of course, it may be argued from a scientific point of view that a part of this chlorine would be taken up from the body itself from contact with the elements in the body, but that would not disprove the fact that it is carried in that way.

Dr. Raugh: Do you just take the solution of chloride of sodium applied on cotton, and touch the electrode to that?

Dr. Rosenthal: The more of the solution you put into the tooth the greater the volume of chlorine. You take a very loose piece of cotton, so it will carry up considerable with it; then if you can get a direct contact you can wrap a little around the electrode also, and saturate that with the chloride of sodium.

Dr. Junkerman: I am familiar with this method of Dr. Rosenthal's, and I think it a very pretty method of treating pulpless teeth, but from my investigation of it, which has been very slight, I am not prompted to go quite so far as Dr. Rosenthal. I think his method is a great deal more simple than he would like to have us understand. For instance, he says he carries by electricity, by means of osmosis, the medicament into the putrescent pulp. If the Doctor will excuse me, I will say that I do not believe he does anything of the kind. His method is just this: He takes sodium chloride and brings it to the part he wishes to affect, and there reduces the sodium chloride into sodium and free chlorine; afterward he turns the chlorine into the chloride of silver and then makes caustic soda, NaOH. He relies upon the element, chlorine, which we all know to be a very strong disinfectant and germicide, to sweeten this putrescent pulp. So far, his method is good, and I know of no more excellent means of purifying anything that is foul than with chlorine gas, and the Doctor deserves a great deal of credit for the introduction of such a simple method. We can all use it in our offices with a cheap arrangement, and control it to the utmost.

He further says that by the introduction of the nitrate of silver he forms in the root canal an insoluble substance known as the chloride of silver. Consequently when he introduces it into the root canal and washes the canal out, he washes out the chloride of silver which may be there.

Furthermore, I do not believe, that by any electrical means, he can carry chlorine gas into a putrescent pulp; he brings it so that it is present and in contact with the putrescent pulp. I have asked him whether it did not work better in putrescent pulps in lower teeth than in the superior ones; he replied no, that it worked equally well in either place. I claim that it will act better in the lower teeth where the gas will fall down, as it can not fall up. As far as saying he fills these root canals with chloride of silver by the introduction of the nitrate of silver, I

do not think he believes that himself, and I do not believe he fills any root canals with the chloride of silver.

Dr. Rees: I would like to ask, will not the 20 percent nitrate of silver discolor the teeth?

Dr. Gensley: So far as the chlorine gas is concerned, of course we all know it is one of the most powerful antiseptics in its nascent state, and it is certainly in that state when decomposed by electricity. I think there would also be some hydrochloric acid generated there; the chlorine would combine with the hydrogen to form the HCl. It is also possible that there would be some ammonia generated in the root canal. Silver chloride is quite soluble in ammonia hydrate.

Dr. Rosenthal: With front teeth in which I have some apprehension of the tooth becoming discolored, I take a piece of cedar wood, whittled down to a fine point, about the size of the root canal; I saturate the piece of wood with a double strength tincture of iodine so as to make it doubly antiseptic. Then I use a preparation of iodoform, which is a method of mine also—for two reasons: I make a salve of iodoform and vaseline, making it thick and heavy, using about two drops of vaseline to the drachm of iodoform. I put this on the end of the little stick, first having notched it, so that it will break off about where I wish. I have not much faith in the antiseptic properties of iodoform, but we will take it for what it is worth, and it oils the root canal. When I use that I do not use the chloride of silver at all, but I simply force the stick in the pulp canal and break it off. Then you may temporarily fill the tooth, but I do not feel the necessity of having any apprehension at all. However, it is an easy matter to take a little pair of plyers and withdraw the stick by reason of the vaseline and iodoform, if it should be necessary.

In the matter of filling the front teeth, I probably should have been a little more explicit in my paper, but I thought these points would come up, and that they would afford subject for discussion. You can not do things in dentistry, or in any other profession, one time as you do at another. The question is asked, would the tooth not turn black? If the chloride of silver were left in a front tooth, where light would strike it, that tooth would undoubtedly turn black, and in these cases I use a stick of cedar wood as indicated. I am very careful to cleanse out all the chloride of silver there is in the cavity, and seal the pulp canal with os-artificial so as to prevent light getting through. To make the tooth look as white as possible, I frequently use the oxyphosphate so as not to allow the gold to show through. However, I should have said that you must dry out the pulp canal and rid it of all the water there is there, leaving simply the dry powder. This can be accomplished by the hot-air syringe, and it takes an incredibly short time to dry it out perfectly. There is chloride of silver deposited from commencement of the root canal to the very apex of the tooth. This is so sufficiently demonstrated that it is not necessary to enter into any argument about it. With a tooth out of the mouth, with the chloride of sodium in the cavity, subsequently when you put the nitrate of silver there and break the tooth open you will see it; there is no doubt about that.

It is no longer the nitrate of silver, but is precipitated into the chloride of silver. I do not attempt first to prepare the cavity for filling; I leave the *debris* all there and cut it out afterward.

I have used this method for over two years now, and I can report to you its success. It takes twenty-five or thirty minutes, but see the time that you save in the end. I invited Dr. Junkerman to criticise the paper, and he has done the best he could under the circumstances. So far as the points he makes with reference to its not being carried through the tooth, the fact that you can take an electrode, as I said before, and place it in a solution of nitrate of silver carrying it right through the tooth and forming the chloride of silver; how does it get there? How is that formed? Is there any electrolysis known that will produce the chloride of silver without the presence of chlorine in nitrate of silver? Can chloride of silver be produced without chlorine? How does it get through the tooth? It would entirely do away with the principle of osmosis. The principle of osmosis would not have a single grain of standing if it were not for its ability of carrying an element from positive to negative. The text-books will tell you that it is probably a mechanical force carrying elements from the anode to the cathode.

The chloride of silver will discolor under the influence of light, and if I were to fill the front teeth with that method and leave free chloride of silver on the walls of the tooth, the light penetrating the tooth would, in time, decompose it and you would have a black precipitate, instead of white, as it was originally.

